# SUMO1 modification of IGF-1R combining with SNAI2 inhibited osteogenic differentiation of PDLSCs stimulated by high glucose

**DOI:** 10.1186/s13287-021-02618-w

**Published:** 2021-10-18

**Authors:** Rongrong Jiang, Miao Wang, Xiaobo Shen, Shuai Huang, Jianpeng Han, Lei Li, Zhiliang Xu, Chengfeng Jiang, Qiao Zhou, Xingmei Feng

**Affiliations:** 1 Department of Stomatology, Affiliated Hospital of Nantong University, 20 Xisi Road, Nantong, 226001 Jiangsu China; 2 Key Laboratory of Neuroregeneration of Jiangsu and Ministry of Education, Nantong University, Nantong, 226001 Jiangsu China

**Keywords:** IGF-1R, Differentiation, Periodontal ligament stem cells, SNAI2, High glucose

## Abstract

**Background:**

Periodontal disease, an oral disease characterized by loss of alveolar bone and progressive teeth loss, is the sixth major complication of diabetes. It is spreading worldwide as it is difficult to be cured. The insulin-like growth factor 1 receptor (IGF-1R) plays an important role in regulating functional impairment associated with diabetes. However, it is unclear whether IGF-1R expression in periodontal tissue is related to alveolar bone destruction in diabetic patients. SUMO modification has been reported in various diseases and is associated with an increasing number of biological processes, but previous studies have not focused on diabetic periodontitis. This study aimed to explore the role of IGF-1R in osteogenic differentiation of periodontal ligament stem cells (PDLSCs) in high glucose and control the multiple downstream damage signal factors.

**Methods:**

PDLSCs were isolated and cultured after extraction of impacted teeth from healthy donors or subtractive orthodontic extraction in adolescents. PDLSCs were cultured in the osteogenic medium with different glucose concentrations prepared by medical 5% sterile glucose solution. The effects of different glucose concentrations on the osteogenic differentiation ability of PDLSCs were studied at the genetic and cellular levels by staining assay, Western Blot, RT-PCR, Co-IP and cytofluorescence.

**Results:**

We found that SNAI2, RUNX2 expression decreased in PDLSCs cultured in high glucose osteogenic medium compared with that in normal glucose osteogenic medium, which were osteogenesis-related marker. In addition, the IGF-1R expression, sumoylation of IGF-1R and osteogenic differentiation in PDLSCs cultured in high glucose osteogenic medium were not consistent with those cultured in normal glucose osteogenic medium. However, osteogenic differentiation of PDLCSs enhanced after adding IGF-1R inhibitors to high glucose osteogenic medium.

**Conclusion:**

Our data demonstrated that SUMO1 modification of IGF-1R inhibited osteogenic differentiation of PDLSCs by binding to SNAI2 in high glucose environment, a key factor leading to alveolar bone loss in diabetic patients. Thus we could maximize the control of multiple downstream damage signaling factors and bring new hope for alveolar bone regeneration in diabetic patients.

## Introduction

Diabetes mellitus (DM) is one of the most prevalent chronic diseases worldwide, and the prevalence of DM in adults is about 10% [1]. Diabetes mellitus is a metabolic disease characterized by a chronic increase in blood glucose levels, while hyperglycemia is caused by the defect in insulin secretion or impairment of its biological function, or both. The chronic presence of hyperglycemia leads to chronic damage to various tissues, especially the eyes, kidneys, heart, blood vessels, and nerves. Diabetic patients are also more likely to induce chronic periodontal disease, which can lead to tooth loosening, occasional pain and discomfort, impaired masticatory function, and eventually tooth loss [2, 3]. The risk of osteoporosis in diabetic patients would significantly rise, the balance between osteoblasts and osteoclasts is disrupted, and osteogenesis is significantly inhibited [4]. At the clinic, we often find that diabetic patients have gum recession and crown elongation, while X-rays can show horizontal resorption of the alveolar bone between the teeth. Compared to their peers, the teeth of diabetic patients are more likely to loosen and fall out. However, there are segments of common clinical treatment, such as supragingival scaling, subgingival scraping, local rinsing, loose tooth fixation, etc., which are extremely limited. All of these tell us that diabetes is a very important factor in the loss of alveolar bone. Therefore, it is necessary to explore the cause of alveolar bone resorption and find new clinical approaches to achieve alveolar bone regeneration in diabetic patients.

Periodontal ligament is the dense connective tissue that exists between the roots of the teeth and the alveolar bone, extending into the alveolar bone or gingiva at one end and into the bone at the other, serving to anchor the roots and relieve masticatory pressure [5]. Periodontal tissue contains a variety of cells, including fibroblasts, osteoblasts, osteoclasts and odontogenic osteoclasts, which are involved in matrix formation. PDLSCs are isolated and cultured from periodontal tissues, which have self-renewal and differentiation potential and are one of the key cells for periodontal tissue regeneration engineering [6]. So far, the clinical applications of stem cell therapy are very promising, including dental, craniofacial and orthopedic treatments, especially in bone repair and regenerative therapy [7]. Bone marrow mesenchymal stem cells (BM-MSCs) can differentiate into a variety of bone matrix cells, which have been used to treat osteonecrosis of the femoral head caused by glucocorticoids [8]. However, MSCs exhibit degenerative changes during aging, including imbalanced differentiation and reduced proliferation, resulting in age-related bone loss [9]. PDLSCs are self-renewing adult multifunctional stem cells of mesodermal origin, which can differentiate into osteoblasts, chondrocytes, adipocytes and nerves. Stem cells have been used to treat a variety of diseases, especially in bone defects [10]. PDLSCs are more accessible and have greater activity and proliferation capacity than other MSCs such as BM-MSCs and UC-MSCs, therefore it is of great importance to study the specific mechanisms of osteogenic differentiation of PDLSCs, which widely presented in periodontal tissues.

Insulin-like growth factor 1 receptor (IGF-1R) expression increased in diabetic patients and also abnormally increased in diabetic osteoporosis rat models [11]. IGF-1R mainly exists on the cell membrane and can be activated by insulin-like growth factors, causing phosphorylation of its own tyrosine kinase structural domain, initiating intracellular signaling, and regulating cell growth and differentiation [12]. Membrane proteins have multiple functions and play an important role in cell recognition, cell signaling, and transport, which are excellent drug targets. Post-translational modifications (PTMs) are post-translational chemical modifications of proteins, and PTMs are complex processes that involve in almost all cellular life processes, which play an extremely important regulatory role. It has more than 400 kinds, mainly including phosphorylation, glycosylation, acetylation, ubiquitination, sumoylation and some other niche modifications [13]. However, high glucose significantly upregulates the expression of PIASy, SUMO1 and SUMO2/3 in a dose and time dependent manner, inducing IKKγ phosphorylation and sumoylization, which then triggers nuclear factor kappa-B (NF-κB) protein signaling [14]. It has been shown that IGF-1R could be sumoylated by IGF-1 stimulation and translocate to the nucleus to act as a transcriptional cofactor and then play a biological role [15]. In the serotype model of human corneal epithelial cells, IGF-1R not only mediates the signal transduction on the plasma membrane, but also can be sumoylated through SUMO1, and transport to the nucleus under the action of IGF-1 [16]. Therefore, IGF-1R is of great study value in the consideration of the etiology of periodontal disease in diabetic patients.

Snail Family Transcriptional Repressor 2 (SNAI2) is widely present in stem cells and essential for osteoblast differentiation, which is an osteogenic-related protein-coding gene [17–19]. Also, this osteogenic stem cell factor is associated with the transcriptional regulation of stem cell genes and proteins activated during osteogenic differentiation [20]. In addition, SNAI2 binds to the RUNX2 promoter to promote osteogenic differentiation [21]. Its related pathways include direct p53 effector and Hippo signaling pathway. SNAIl/SNAI2 can also regulate stem cell function by forming a complex with the transcriptional coactivators YAP and TAZ, inhibiting Hippo pathway-dependent regulation of the YAP/TAZ signaling cascade. In turn, the SNAI1/SNAI2 axis activates a series of downstream targets of bone formation [22]. In conclusion, these results suggest that the search for the key of upstream and downstream regulation of this transcription factor, SNAI2, during osteogenic differentiation of stem cells remains a hot topic of study.

In order to investigate the causes of progressive alveolar bone loss in diabetic patients, PDLSCs were isolated from healthy individuals after extraction of interrupted teeth or orthodontic reduction in adolescents, and vitro models were established with different glucose concentrations to simulate high glucose environment. Combining with the current status of diabetes research, we want to investigate the reasons for IGF-1R inhibition of osteogenic differentiation of PDLSCs in high glucose environment. And the effects of different glucose concentrations on the osteogenic differentiation ability of PDLSCs were investigated at the genetic and cellular levels using staining assay, Western Blot, RT-PCR, Co-IP and cytofluorescence. This study will further enrich the pathogenesis of periodontal diseases caused by diabetic patients and provide a theoretical basis for finding therapeutic targets for periodontal diseases in diabetic patients.

## Materials and methods

### Separation, culture of PDLSCs

The culture procedure of PDLSCs was performed according to the previous research method of our group [23] and was approved by the ethics committee of the Affiliated Hospital of Nantong University. Ten orthodontic premolars and impacted teeth from patients aged 14–22 years were collected from the outpatient clinic of the Department of Dentistry, after obtaining informed consent from all patients and parents of adolescents. All participants rinsed their teeth with sodium bicarbonate solution before extraction, and immediately the teeth were placed in the phosphate-buffered saline (PBS) containing 100 U/ml penicillin and 100 μg/ml streptomycin (Invitrogen, Life Technologies, Carlsbad, CA). The periodontal tissue was treated with a sterile blade in an ultra-clean table. The periodontal membrane tissue was scraped from the middle third of the tooth surface in the ultra-clean table and digested in 3 mg/ml type I collagenase (Worthington Biochem, Freehold, NJ) and 4 mg/ml dispase (Roche, Mannheim, Germany) for 30 min. Cell suspensions of periodontal membranes were inoculated into 25 cm cell culture dishes and suspended in complete medium containing low glucose Dulbecco's modified Eagle's medium (GIBCO BRL, Grand Island, NY) with 10% fetal bovine serum (FBS), 100 U/ml penicillin and 100 μg/ml streptomycin. Cells were inoculated in tissue culture dishes and cultured at 37 °C with 5% CO_2_, and the medium was changed every 3 days. When the cells in the culture dish reached 80%, they were subcultured in a ratio of 1:3. So the experiments were performed with the fourth generation of periodontal stem cells.

### Osteogenic differentiation

The osteoblast differentiation process was performed as described in our previous study [24]. The P4 generation of PDLSCs were used as the study subject and inoculated in 6-well culture plates at a density of 3000 cells/cm^2^, and the osteogenic differentiation induction medium (50 µg/mL ascorbic acid, 10 mM sodium glycerophosphate, and 1.0 µg/mL dexamethasone) was configured with different glucose concentrations of complete culture medium (5. 5 mmol/L, 11 mmol/L, 25 mmol/L, 44 mmol/L), 11 mmol/L and 44 mmol/L glucose concentrations were configured from L-DMEM with appropriate amount of medical 5% sterile glucose solution, and differentiation induction cultures were incubated for 7, 14 and 21 days, with fluid changes every two days.

### Alizarin red staining and ALP staining

Cells were co-cultured with osteogenic medium for 21 days and stained to detect the mineralization potential of the cells. The medium in the 6-well culture plate was first discarded, the cells were washed 3 times with appropriate amount of 0.01 M PBS, inverted to drain the remaining water, and the cells were fixed with 4% paraformaldehyde for 30 min. The cells were then washed 3 times with an appropriate amount of 0.01 M PBS and the remaining water was drained. According to the manufacturer's instructions, the cells were then incubated with 40 mM Alizarin Red S solution (Solarbio) for 15 min for Alizarin Red staining and with 40 mm Alkaline Phosphatase Assay Kit (Beyotime) for 12 h for Alkaline Phosphatase staining.

### Western blot

The cellular protein extraction process was performed as described in our previous study [25]. Cells cultured in osteogenic induction medium with different glucose concentrations were first fully lysed using PMSF solution and RIPA solution (Beyotime), followed by protein collection and centrifugation to extract the supernatant. Membrane, Nuclear and Cytoplasmic Protein Extraction kit (Sangon Biotech, C510002) were used to extract membrane and nuclear proteins from cells cultured in osteogenic medium with different glucose concentrations according to the product instructions, and then the protein concentration was measured by BCA method. The protein concentrations were then measured by BCA to sample the calculated amounts. After 90 min, the PVDF membrane was placed in the closure solution and gently shaken on a shaker for 2 h at room temperature. The primary antibody was incubated overnight at 4 °C and the secondary antibody was incubated for 2 h at room temperature. The following primary antibodies were used: rabbit anti-SNAI2(1:1000,Cell Signaling Technology), rabbit anti-RUNX2 (1:1000, Sangon Biotech), rabbit anti-IGF-1R (1:1000, Abcam), rabbit anti-β-actin (1:1000, Abbkine), mouse anti-SUMO1 (1:1000, Santa Cruz),mouse anti-PCNA (1:1000, Santa Cruz),mouse anti-PI3K (1:1000, Santa Cruz), mouse anti-pAKT (1:1000, Santa Cruz), rabbit anti-AKT (1:1000, Proteintech), and rabbit anti-Na^+^-K^+^-ATPase (1:1000, Abbkine). The second antibodies were goat-anti-rabbit or goatanti-mouse horseradish peroxidase-conjugated IgG (1:1500, Abcam).

### Real-time polymerase chain reaction analysis

According to the manufacturer's instructions (Invitrogen), total RNA was extracted from cells by Trizol reagent. Complementary DNA (cDNA) miRNA transcripts were amplified by Maxima™ H Minus cDNA Synthesis Master Mix, with dsDNase (Thermo Fisher Scientific, Waltham, MA). The polymerase chain reaction (PCR) mixture was prepared by LightCycler 480 SYBR Green (Roche Applied Science, Penzberg, Germany). For β-actin, RUNX2 and SNAI2 detection, cDNA was synthesized by RevertAid RT Reverse Transcription Kit (Thermo Fisher Scientific). AceQ qPCR SYBR Green Master Mix (without ROX) (Vazyme) was then used for quantitative PCR of these genes. GAPDH was used for normalization. We used a Light Cycler 480 Real-Time PCR System (Roche Diagnostic, Mannheim, Germany) to test these levels. The primer sequences used in the experiment were as follows: β-actin:5′-TTAATAGTCATTCCAAATATGA-3′,5′-GGGACAAAAAAGGGGGAAGG-3′;RUNX2:5′-TCAACGATCTGAGATTTGTGGG-3′,5′-TCAACGATCTGAGATTTGTGGG-3′;SNAI2:5′-TGTGACAAGGAATATGTGAGCC-3′,5′-TGAGCCCTCAGATTTGACCTG-3′.

The ratio obtained by comparing the target gene with the CT value is the relative expression of the gene. Three replicates were set up in each experiment, three times in total.

### Co-immunoprecipitation

Co-immunoprecipitation protocol was performed by using a Pierce co-IP kit (Thermo Scientific™ Pierce™ Classical magnetic bead immunoprecipitation/immunocoprecipitation Kit, 88804). Incubate the cell lysate with the rabbit anti-IGF-1R (Abcam) for 1–2 h at room temperature, or overnight at 4 °C. Bind the antigen/antibody complex to the protein A/G magnetic beads for one hour at room temperature. Wash the beads twice with immunoprecipitation lysis/rinse buffer, followed by one wash with pure water. Elute the antigen/antibody complexes. Finally, immunoblotting experiments for immune complexes were performed.

### Immunofluorescent staining

PDLSCs were seeded into 24-well plates with a density of 1 × 10^5^cells/mL. When the cells were growing well, we added osteogenic induction solution until 36 h. Then we fixed the cells with 4% paraformaldehyde 30 min, rinsed with PBS containing 0.1% Trito X‐100 (PBST), and blocked for 30 min in PBST supplemented with 10% FBS. Cells were incubated with the primary antibody overnight at 4 °C. Then the cells were rinsed and incubated with secondary antibodies for 2 h at room temperature. The primary antibody was diluted in proportion with Immunostaining Blocking/Primary Antibody Dilution Buffer (Sangon Biotech) and incubated overnight at 4 °C. PDLSCs were incubated with the corresponding secondary antibody for 2 h at room temperature. Nuclei were stained with DAPI (1:1000; Santa Cruz) and the cells were mounted on an inverted fluorescent microscope and photographed.

### CCK-8 assay

PDLSCs were seeded on 96-well plates at a cell density of 1 × 10^3^ cells/well. 0, 0.5, 1, 2, 5, 10, 20, 50umol/L IGF-1R inhibitor NVP-ADW742 (Beyotime, Shanghai, China) were added into Osteogenic Induction Solution and were used to culture PDLSCs for 24 h, 48 h, 72 h. Then we washed the wells, added 100 uL L-DMEM and 10 uL CCK8 (Beyotime, Shanghai, China) per well, and incubated the cells at 37 °C for 2 h. The optical density was measured at 450 nm.

### Statistical analysis

The experimental data were analyzed by the GraphPad Prism software. All experiments were repeated at least three times, with three replicate wells per design and the data shown as mean ± standard deviation. Statistical significance was assessed by one-way ANOVA analysis of variance and minimum significant difference test (Fisher’s least significant difference [LSD]). *p* < 0.05 was considered statistically significant, and *p* < 0.01 and *p* < 0.001 were considered as highly significant. All statistical analyses were performed using SPSS 23.0 (IBM Corp., USA).

## Results

### High glucose environment inhibited osteogenic differentiation of PDLSCs

**Fig. 1:**
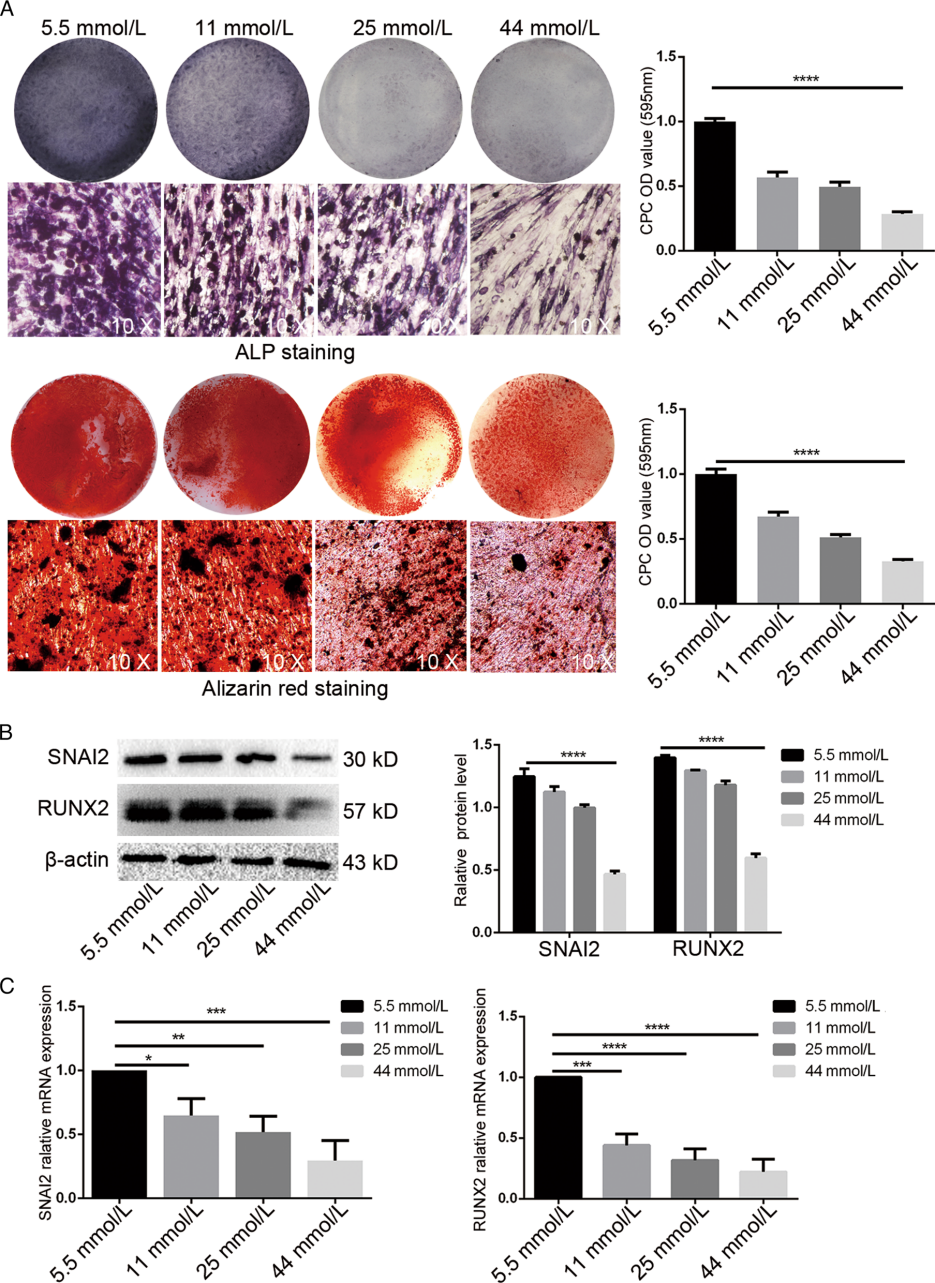
Osteogenic differentiation of PDLSCs under osteogenic medium with different glucose concentrations. PDLSCs were cultured in osteogenic medium containing glucose at concentrations of 5.5 mmol/L, 11 mmol/L, 25 mmol/L, 44 mmol/L. **a** Osteogenic differentiation was determined by alizarin red staining and ALP staining after 21 days; Quantitative measurement of mineralization, and the absorbance was measured at 595 nm by spectrophotometry (****p < 0. 0001); **b** RUNX2 and SNAI2 was measured by Western Blot at 14 days of osteogenic induction. β-actin as an internal reference. And Quantitative analysis of expression levels (****p < 0. 0001); **c** the expression of RUNX2 and SNAI2 in PDLSCs were evaluated by RT-PCR at 7 days of osteogenic induction. The data are expressed as the mean ± SD of at least three replicates, *p < 0. 05; **p < 0. 01; ***p < 0. 001; ****p < 0. 0001, all results were obtained from at least three independent experiments. *PDLSCs* periodontal ligament stem cells, *RT-PCR* real-time quantitative polymerase chain reaction, *SD* standard deviation

In order to investigate the effects of glucose concentrations on osteogenic differentiation of PDLSCs, different concentrations of glucose (5.5 mmol/L, 11 mmol/L, 25 mmol/L and 44 mmol/L) were added to the osteogenic medium and were used to grow PDLSCs. Then we analyzed by using alizarin red staining and ALP staining. The results showed that 21 days later, compared with 5.5 mmol/L osteogenic medium, the amount of mineralized nodule formation of PDLSCs gradually decreased by means of concentration dependent, and the osteogenic differentiation ability of PDLSCs decreased (Fig. 1a) as glucose concentration increased. The osteogenic differentiation transcription factor RUNX2 is a key protein involved in osteoblast differentiation and bone morphogenesis, which is essential for osteoblast maturation as well as intra-membrane and endochondral ossification. SNAI2 is present in stem cells and is essential for osteoblast differentiation. Also, SNAI2 is associated with the transcriptional regulation of stem cell genes and genes activated during osteogenic differentiation. In addition, SNAI2 also binds to the RUNX2 promoter to promote osteogenic differentiation. The protein levels of SNAI2 and RUNX2 were examined by Western Blot after 14 days of osteogenic induction in PDLSCs at different glucose concentrations. The results similarly showed that the level of RUNX2 in PDLSCs at 14 days of osteogenic induction gradually decreased in a glucose concentration-dependent manner. Meanwhile SNAI2 also gradually decreased (Fig. 1b). These indicated that high glucose significantly inhibited the osteogenic differentiation of PDLSCs. Subsequently, the mRNA expression of SNAI2, RUNX2 in PDLSCs was examined by RT-PCR after 7 days of osteogenic differentiation induction by different glucose concentrations. The results similarly showed that the mRNA expression of SNAI2, RUNX2 decreased significantly with increasing glucose concentration in the osteogenic medium (Fig. 1c). All of them indicated that high glucose inhibited the osteogenic differentiation of PDLSCs, and SNAI2 was proportional to the osteogenic differentiation of PDLSCs, which suggested that SNAI2 may be involved in the osteogenic differentiation of PDLSCs.

According to our research results and many existing scientific research literature references, we selected 5.5 mmol/Land 25 mmol/L glucose concentration as normal group and high glucose group, respectively, for follow-up experiments. These results suggested that high glucose environment inhibited the osteogenic differentiation of PDLSCs, and SNAI2 might be involved in this process.

### SUMO1 modification of IGF-1R in PDLSCs increased under high glucose environment

**Fig. 2:**
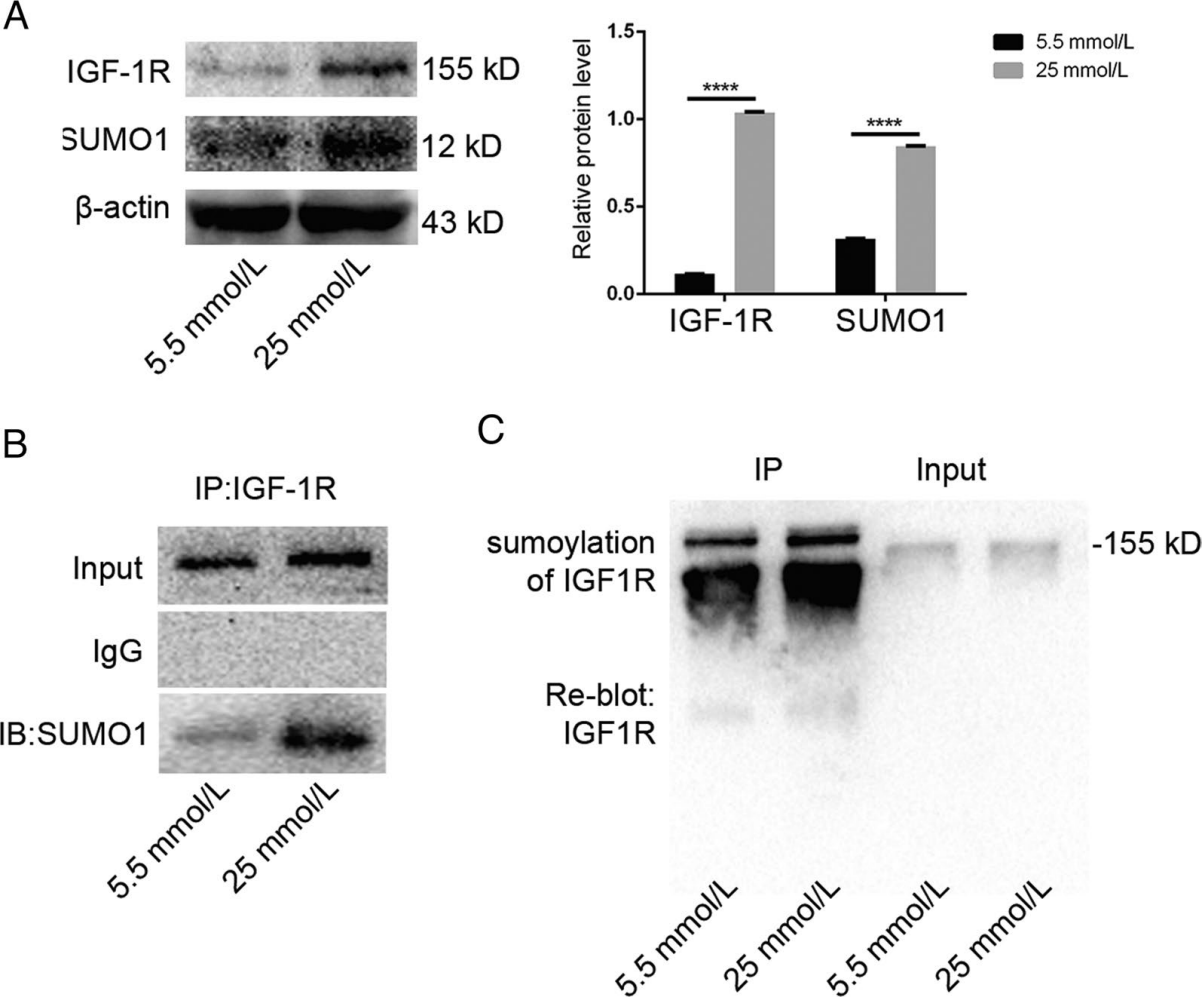
High glucose promoted sumoylation of IGF-1R. 5.5 mmol/L and 25 mmol/L groups were selected for follow-up experiment. **a** IGF-1R and SUMO1 was measured by Western Blot at 7 days of osteogenic induction. β-actin as an internal reference. And Quantitative analysis of expression levels (****p < 0. 0001). **b** Sumoylation of IGF-1R was detected by immunoprecipitation (IP) with anti-IGF-1R antibody followed by Western Blot with anti-SUMO1 antibody. IGF-1R was conjugated with SUMO1 in PDLSCs. **c** Sumoylation of IGF-1R were determined by IP of IGF-1R and IB for IGF-1R. The data are expressed as the mean ± SD of at least three replicates, *p < 0. 05; **p < 0. 01; ***p < 0. 001; ****p < 0. 0001, all results were obtained from at least three independent experiments

Based on the above results, it indicated that osteogenic medium with different glucose concentrations inhibited osteogenic differentiation of PDLSCs in a concentration-dependent manner. Next, we wanted to find the upstream damage factors affecting osteogenic differentiation of PDLSCs in high glucose environment. So we selected the concentration of 5.5 mmol/L for the normal group and 25 mmol/L for the high glucose group for the osteogenic induction of PDLSCs for subsequent experiments. Secondly, we wanted to confirm the relationship between SNAI2 and the decreased osteogenic differentiation ability of PDLSCs under high glucose stimulation, as well as the upstream regulatory factors of SNAI2. The activation of IGF-1R regulates and maintains many key signaling pathways of cell homeostasis, including cell survival, growth, proliferation and differentiation. The imbalance of sumoylation is related to different disease. Meanwhile, SUMO1 is associated with nuclear translocation and accumulation of IGF-1R. Compared with the normal group, the protein levels of IGF-1R and SUMO1 in PDLSCs induced by high glucose significantly increased after 7 days, it indicated that the degree of sumoylation in PDLSCs was also enhanced (Fig. 2a). In cell models of other diseases, IGF-1R and SUMO1 have been studied thoroughly. However, the osteogenic differentiation ability of stem cells has not been studied, especially in the etiology of diabetic osteoporosis. Recent studies have shown that sumoylation occurs in IGF-1R, which induces nuclear accumulation of IGF-1R [26]. Therefore, we speculate that high glucose stimulation may promote the sumoylation of IGF-1R on cell membrane. SUMO1 modification occurs at three evolutionarily conservative lysine residues of the β-subunit of IGF-1R. Therefore, after 7 days of osteogenic induction, we used anti-IGF-1R for immunoprecipitation and detected SUMO1 by Western Blot. The results showed that the expression in the bands of SUMO1 bound to IGF-1R was significantly richer in the high glucose group than in the normal group (Fig. 2b). Further experiments showed that sumoylation of IGF-1R was detected on the immunoblot. Interestingly, it was found that the blot of IGF-1R had obvious tailing phenomenon (Fig. 2c). In conclusion, these data indicated that sumoylation of IGF-1R were induced by high glucose in PDLSCs.

### Sumoylation of IGF-1R inhibited osteogenic differentiation of PDLSCs by binding to SNAI2 through nucleus entry

**Fig. 3:**
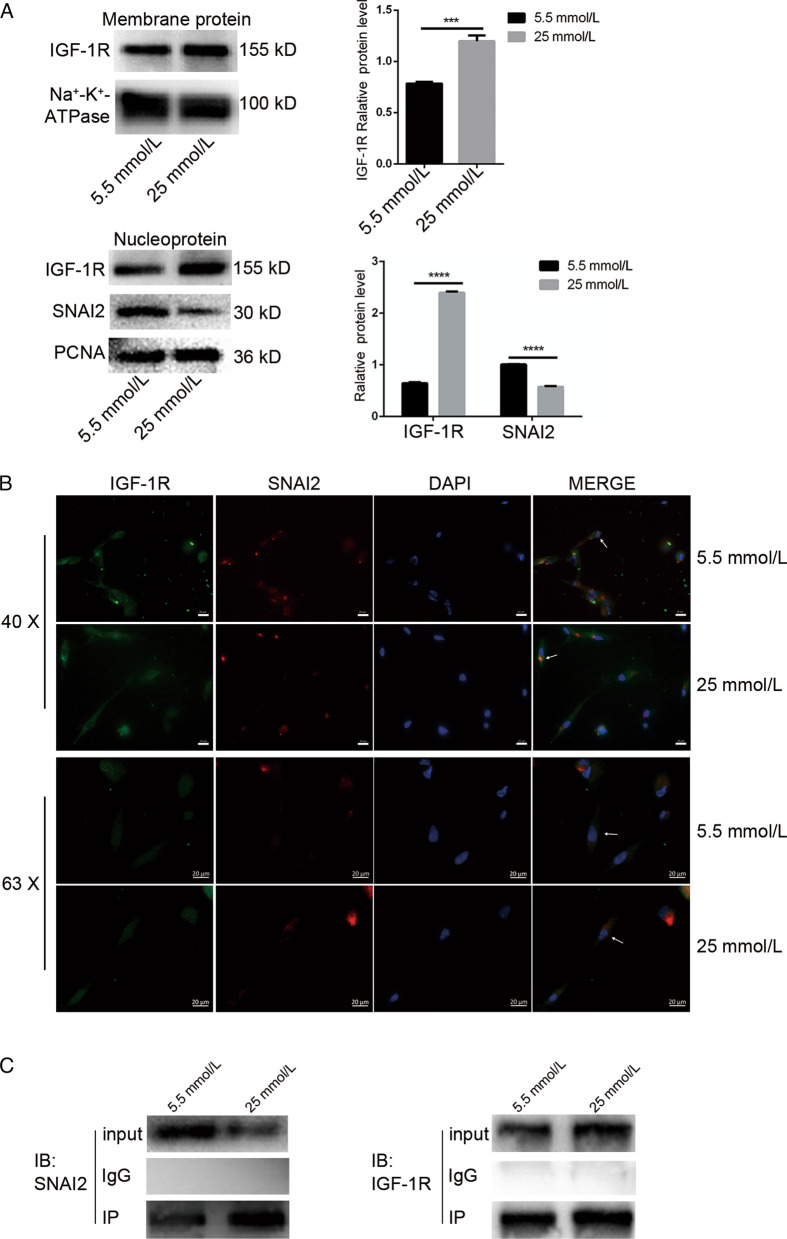
Sumoylation of IGF-1R inhibited osteogenic differentiation of PDLSCs. **a** The level of IGF-1R and SNAI2 were detected by Western Blot in membrane protein and nuclear protein after 7 days of osteogenic induction. Na^+^–K^+^-ATPase and PCNA as an internal reference. And Quantitative analysis of expression levels (***p < 0. 001; ****p < 0. 0001). **b** The expression of IGF-1R (green) and SNAI2 (red) in PDLSCs were examined by cytofluorescence. Nuclei are stained with DAPI (blue). Scale bar 20 μm. **c** IGF-1R was conjugated with SNAI2 in PDLSCs was detected by immunoprecipitation (IP) with anti-IGF-1R antibody followed by Western Blot with anti-SNAI2 antibody. All results were obtained from at least three independent experiments

As mentioned above, IGF-1R and the degree of sumoylation in PDLSCs increased stimulated by high glucose. In order to explore whether the change of IGF-1R induced by high glucose was the key factor affecting the osteogenic differentiation of PDLSCs, we isolated and extracted the membrane protein and nuclear protein. Western Blot showed that IGF-1R in both membrane protein and nuclear protein increased in PDLSCs cultured with high glucose, but SNAI2 in nucleus decreased (Fig. 3a). These told us that sumoylation of IGF-1R might enter the nucleus and then bind to SNAI2, which coincided with the inhibition of osteogenic differentiation of PDLSCs in high glucose environment. To further assess the effect of high glucose on IGF-1R and intranuclear SNAI2 after nuclear accumulation, we performed cytofluorescence for IGF-1R and SNAI2. We found that the expression of IGF-1R increased and the expression of SNAI2 decreased in the nucleus of PDLSCs cultured with high glucose, which was consistent with our previous immunoblot analysis. More interestingly, we found increased nuclear localization of IGF-1R and SNAI2 in the nucleus of PDLSCs cultured in high glucose (Fig. 3b). To further verify the interaction of IGF-1R and SNAI2 in the high glucose environment, we performed immunoprecipitation with anti-IGF-1R antibody after the cells were cultured until 7 days. First, we analyzed the binding of IGF-1R to SNAI2 by immunoprecipitation, and found it enhanced when stimulated by high glucose (Fig. 3c). IGF-1R also increased at the target molecular weight, which was consistent with the previous results (Fig. 2a). From these data, it was shown that high glucose stimulated sumoylation of IGF-1R and binding to SNAI2 after entering the nucleus inhibited the osteogenic differentiation of PDLSCs.

### IGF-1R inhibitor reversed the inhibition of osteogenic differentiation induced by high glucose

**Fig. 4:**
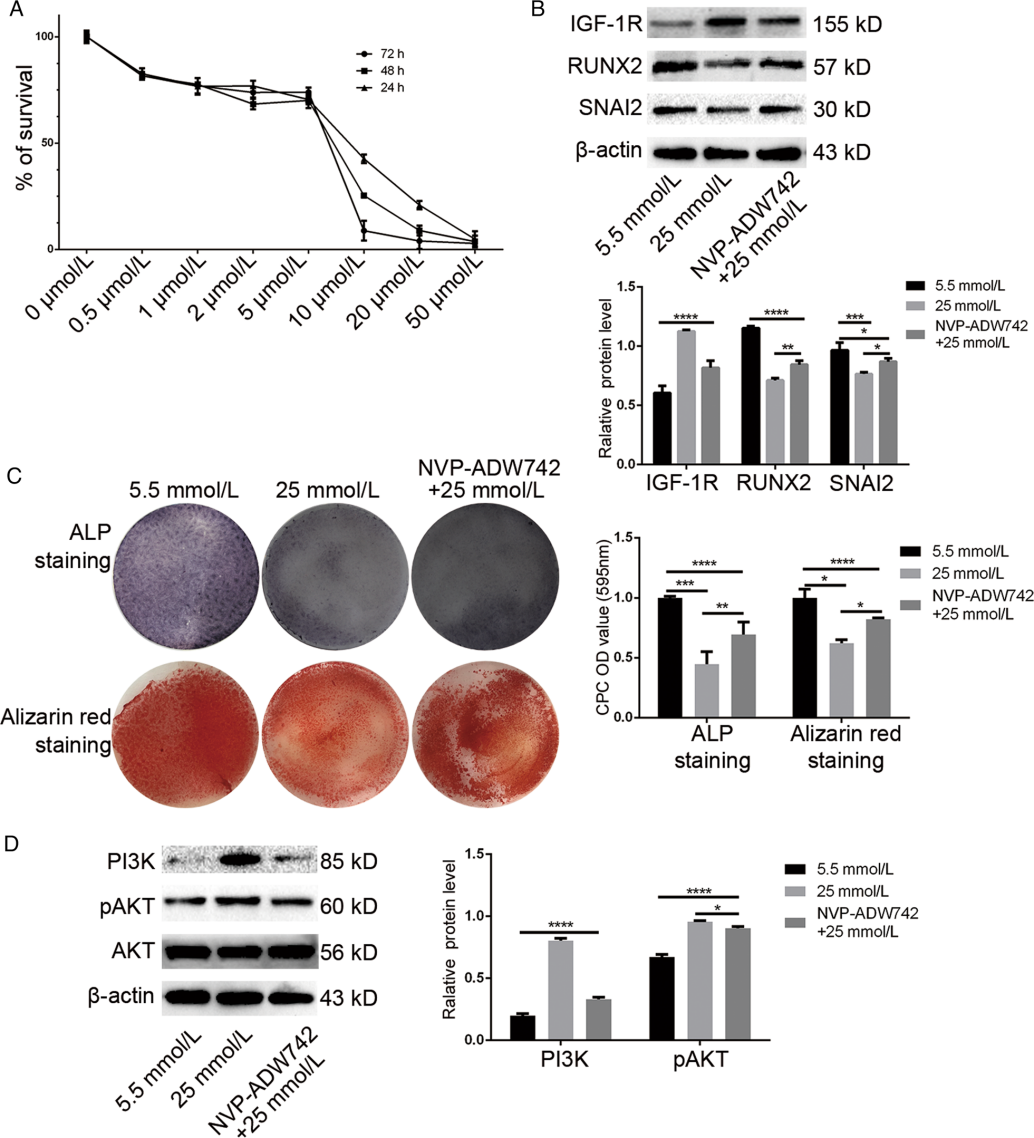
Inhibition of IGF-1R in high glucose environment promoted osteogenic differentiation of PDLSCs. **a** The effects of NVP-ADW742 treatment on cell survival rate were assayed by CCK-8 assay. Cells were cultured for 24 h, 48 h, and 72 h in the concentrations of NVP-ADW742, from 0 to 50 umol/L. The results were obtained from at least three independent experiments. **b** IGF-1R, RUNX2 and SNAI2 were measured by Western Blot at 7 days of osteogenic induction. β-actin as an internal reference. **c** Osteogenic differentiation was determined by alizarin red staining and ALP staining after 21 days; Quantitative measurement of mineralization, and the absorbance was measured at 595 nm by spectrophotometry; **d** Western Blot was performed and found that NVP-ADW742 selectively inhibited the PI3K/AKT signing pathway, which related to the level of IGF-1R. All results were obtained from at least three independent experiments. PI3K, phosphatidylinositol-3-kinase; AKT, protein kinase B. The data are expressed as the mean ± SD of at least three replicates, *p < 0. 05; **p < 0. 01; ***p < 0. 001; ****p < 0. 0001, all results were obtained from at least three independent experiments

To further demonstrate whether IGF-1R played a big role in the inhibition of osteogenic differentiation of PDLSCs in high glucose, we used IGF-1R inhibitor NVP-ADW742 to block the downstream signaling pathway in the subsequent experiments. In recent years, NVP-ADW742 has been used as IGF-1R inhibitor in many studies [27]. Firstly, we performed CCK-8 assay to clarify the effect of NVP-ADW742 concentration on the toxicity and survival of PDLSCs. Cells were collected after 24 h, 48 h and 72 h. CCK-8 assay prompted us to select a concentration of 5 umol/L NVP-ADW742 for subsequent experiments (Fig. 4a). After stimulation with 5 umol/L NVP-ADW742 for 72 h, the level of IGF-1R in PDLSCs was detected, and it was found that the expression level of IGF-1R indeed decreased (Fig. 4b). Then after the medium of NVP-ADW742 group was changed to high glucose osteogenic medium, we continued culturing until we performed Western Blot 7 days later. It was found that the level of RUNX2and SNAI2 in NVP-ADW742 group was slightly higher than that in high glucose (Fig. 4b). Similarly, the medium of NVP-ADW742 group was replaced with high glucose osteogenic medium and the culture was continued until we performed alizarin red staining and ALP staining 21 days later, and the mineralization results were consistent with the Western Blot in Fig. 4b (Fig. 4c). These findings suggested us that IGF-1R inhibitor NVP-ADW742 alleviated the inhibition of osteogenic differentiation caused by high glucose, but the osteogenic mineralization capacity was still inferior to that of PDLSCs cultured in the normal glucose. Thus, it seemed that the high expression of IGF-1R was indeed a key factor leading to the inhibition of osteogenic differentiation. Genetic studies have shown that the IGF-1R/PI3K/AKT signal transduction pathway was involved in a variety of animals, including nematodes, Drosophila and mammals, and linked to many diseases [28]. Recently PI3K/AKT and its downstream signaling pathways have received increasing attention in osteogenic differentiation. To investigate the downstream signaling pathway of IGF-1R inhibiting osteogenic differentiation of PDLSCs, we performed Western Blot on three groups of proteins, then found that IGF-1R activated PI3K/AKT signaling pathway and the activation of PI3K, pAKT was positively correlated with IGF-1R (Fig. 4d). So we suspected that the PI3K/AKT signaling pathway downstream of IGF-1R plays the same role in inhibiting osteogenic differentiation of PDLSCs in high glucose.

## Discussion

**Fig. 5:**
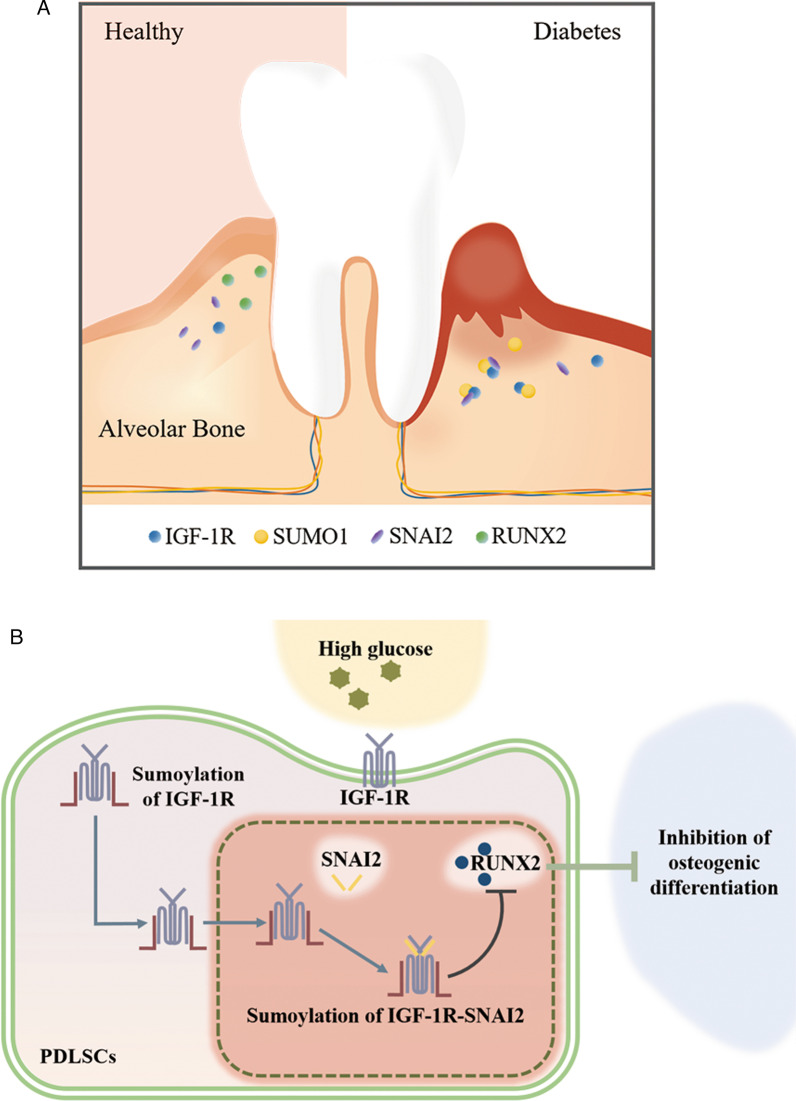
The mechanism of IGF-1R inhibiting osteogenic differentiation of PDLSCs stimulated by high glucose. **a** Under normal condition, the regenerative function of the alveolar bone around the teeth is stable and PDLSCs can differentiate into osteoblasts and other multifaceted differentiation. Whereas in diabetic patients, the alveolar bone is gradually resorbed and the teeth are loosened, IGF-1R overexpressed. **b** In this study, osteogenic medium with high glucose concentration were used to culture PDLSCs to mimic the periodontal tissue microenvironment of diabetic patients. Under the stimulation of high glucose, the level of IGF-1R located on the cell membrane significantly increased, along with the enhancement of SUMO modifications. Subsequently, sumolylation of IGF-1R inhibited downstream osteogenic-related proteins by entering the nucleus and combining with SNAI2, resulting in reduced osteogenic differentiation of PDLSCs under high glucose environment. *IGF-1R* the insulin-like growth factor 1 receptor, *SUMO1* Small Ubiquitin Like Modifier 1, *SNAI2* Snail Family Transcriptional Repressor 2, *RUNX2* RUNX Family Transcription Factor 2, *PDLSCs* Periodontal ligament stem cells

The key point of this study was to find a new mechanism for high glucose to inhibit the osteogenic differentiation of PDLSCs (Fig. 5). Bone loss and bone remodeling around adult teeth are regulated by a dynamic balance between osteoclasts and osteoblasts. However, under the influence of glucose, aging, and inflammation, the osteogenesis-related marker protein RUNX2 decreases and leads to limitation of alveolar bone regeneration. Under physiological conditions, bone tissue has reconstructive activity. That is, osteoclasts mediate the resorption of old bone and osteoblasts mediate the formation of new bone [29]. Bone resorption and formation form a balance to keep the total amount of bone tissue relatively stable. This mechanism is also known as bone remodeling [30]. As we all know, osteoporosis is a serious complication of diabetes, which has been the focus of scholars for a long time. Recent studies have reported that the number of osteoblasts in the distal radial trabeculae decreased and the number of osteoclasts increased, and a larger bone gap in the trabecular network in diabetic patients [31]. Diabetes and periodontitis have been considered to be biologically related and high glucose induces oxidative stress, which inhibited osteogenic differentiation of PDLSCs [32]. It has also been found that high glucose induces NF-κB activation and leads to high expression of IL-6 and IL-8, thereby inhibiting osteogenic differentiation of PDLSCs [33]. The osteogenic differentiation ability of PDLSCs was significantly reduced in high glucose, and these findings were consistent with our results. These suggested us that alveolar bone resorption was related to the metabolic imbalance between osteoblasts and osteoclasts in diabetic patients. Therefore, studying the mechanisms of osteogenic imbalance in diabetic patients is of great significance for controlling the progression of periodontal disease. However, we only explored the effect of IGF-1R on osteogenic differentiation of PDLSCs under high glucose stimulation temporarily. In future, we will concentrate on studying the effect of IGF-1R on osteoclasts in periodontal tissues, and perhaps the latter is also of interest.

Previous studies have shown that there were multiple signaling pathways involved in the regulation of osteoblast metabolic homeostasis during osteogenic differentiation, such as the PI3K/AKT signaling pathway that promoted the progression of osteoporosis [34]. IGF-1R is a membrane protein that expresses on the surface of a variety of cells, including hepatocytes, myocytes and osteocytes [35]. In recent years, the role of IGF-1R in osteogenesis and bone reconstruction has received increasing attention. Recent studies have shown that IGF-1R could be involved in alterations in blood glucose levels and bone metabolism [36, 37] and interfered with the osteogenesis-related Wnt/β-catenin signaling pathway [38, 39]. It has also been shown in animal experiments that IGF-1R and phosphorylation of IGF-1R were also significantly higher in OVX + streptozotocin-induced osteoporosis model rats than in normal rats [34]. Besides, IGF-1R nuclear transfer has been fully studied in many other fields, such as oncology and ophthalmology [40]. Our research further shown that the nuclear transfer of IGF-1R in high glucose environment was mediated by SUMO modification. More interestingly, sumoylation of IGF-1R nuclear translocation bound to SNAI2 in the nucleus and thus inhibited downstream osteogenic related proteins (Fig. 5b). These have never been studied in the aspect of stem cell osteogenesis differentiation. These findings may provide an effective method for the treatment of periodontal disease in diabetic patients.

Sumoylation of proteins is an important regulatory element regulating protein function in the human body, which is involved in the pathogenesis of a variety of human diseases. More and more evidence have shown that sumoylation played important roles in the pathogenesis of diabetes [41]. However, we unexpectedly found that sumoylation of IGF-1R played a significant role in the process of stem cell osteogenic differentiation, especially in regulating downstream osteogenic-related transcription factors such as SNAI2 and RUNX2. SNAI2 regulated the self-renewal and osteogenic differentiation potential of bone marrow mesenchymal stem cells [42]. Secondly, we also discovered that IGF-1R inhibitors significantly reversed the inhibition of osteogenic differentiation stimulated by high glucose after adding appropriate amounts of NVP-ADW742 in high glucose osteogenic medium. Thus IGF-1R and its downstream signaling axis may play a role in inhibiting osteogenic differentiation of stem cells, as similarly demonstrated in our study. However, the role of post-translational modification of IGF-1R in high glucose deserves further investigation, such as the phosphorylation of IGF-1R, and the protein modification makes IGF-1R structure more complex and makes the function more perfect.

However, the etiology of periodontal disease is complex. For diabetic patients, endocrine disorders, dietary and nutritional aspects, and the long-term use of medications may be associated with progressive resorption of the alveolar bone. Therefore there are still a lot of work to be done to solve the periodontal problems in diabetic patients. It is necessary to collect periodontal tissues from diabetic patients after tooth extraction for proteomic analysis and RNA sequencing to further study, which will be the focus of human attention in the future.

## Conclusion

In summary, our data demonstrated that IGF-1R inhibited the osteogenic differentiation of PDLSCs via combining with SNAI2 stimulated by high glucose. Therefore, IGF-1R might be a molecular target to regulate the osteogenic differentiation for therapeutic agents in dental medicine. However, whether IGF-1R is involved in the formation of osteoclasts in periodontal tissues needs to be further investigated.

## Data Availability

The datasets used and/or analyzed during the current study are available from the corresponding author on reasonable request.
